# Supplementary figures and images for: Random forest algorithms for recognizing daily life activities using plantar pressure information: a smart-shoe study
Source: PeerJ. 2020 Oct 28;8:e10170. doi: 10.7717/peerj.10170 (PMC7602692; doi:10.7717/peerj.10170)

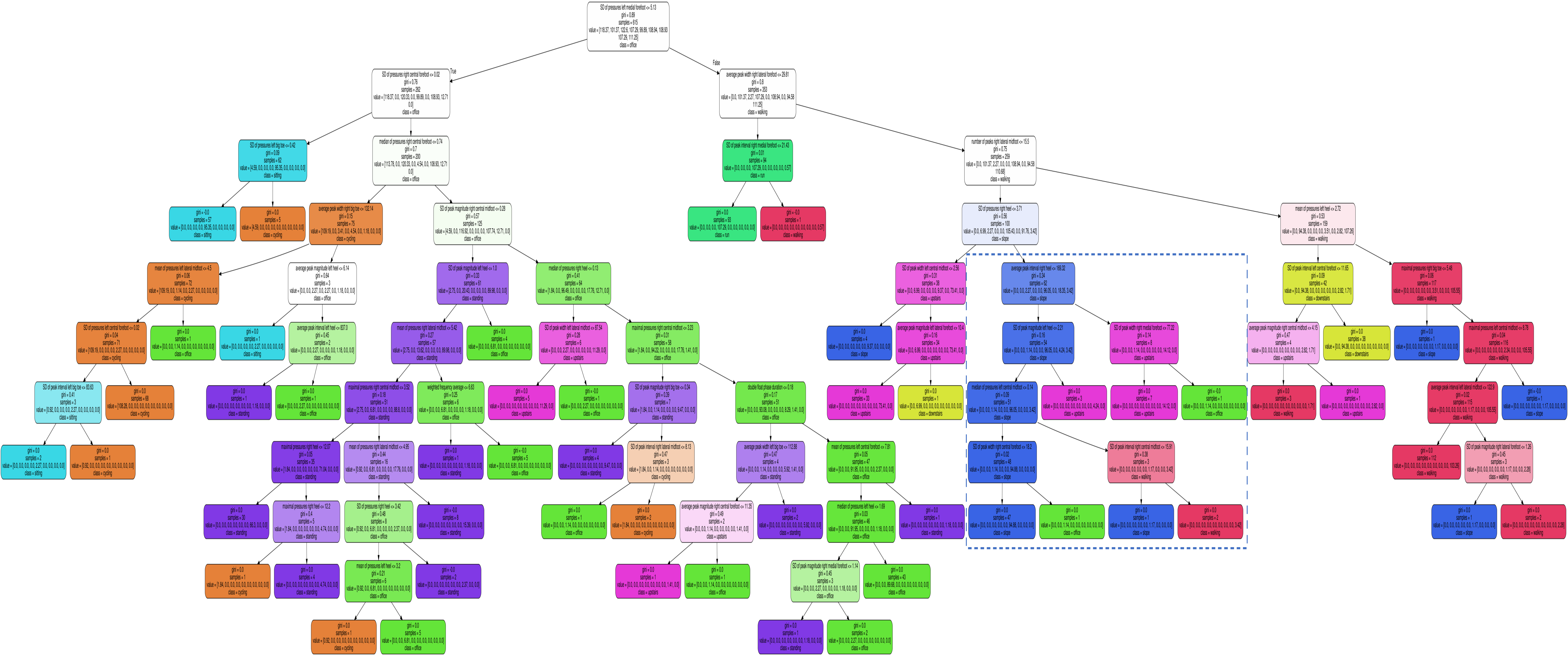

Supplement: Supplemental Information 1 — The tree is composed of 101 nodes and leaves. This number may vary from trees to tress. During the training process, nodes are split until all data points correspond to one activity. At each node, the decision is based on the parameter that best discriminates the sample in two sub-samples. The process is repeated until the generation of pure offspring, i.e. leaves containing data points for one given activity only. Gini: sample impurity, score from 0 to 1, with 0 indicating pure offspring. Samples: number of data point evaluated by the nodes. Value ([cycling, downstairs, office, run, sitting, slope, standing, upstairs, walking]): weight of each activity in the evaluated sample; 0 indicates the absence of data point for one given activity. Class: activity with the most data point. The tree is extracted from the following forest: window length: 20 sec, configuration: 7 sensors, assignment: 1, run: 1. [file peerj-08-10170-s001.png]

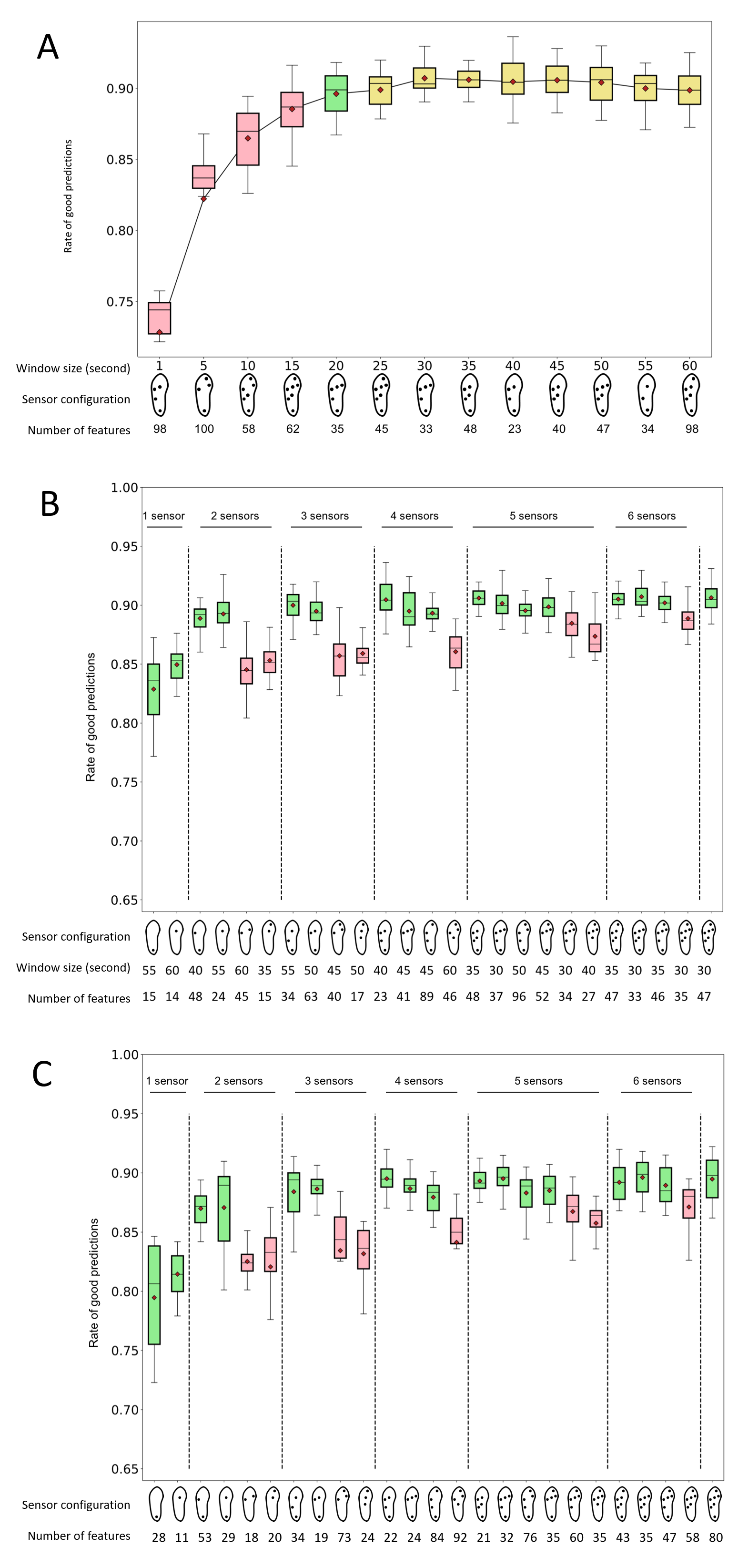

Supplement: Supplemental Information 4 — (A) window length effect on activity recognition rate (best combination for “number and location of sensors” and “number of features”). Pink boxes: 1, 5, 10 and 15 seconds. Green box: 20 seconds (considered optimum). Yellow boxes: 25, 30, 35, 40, 45, 50, 55 and 60 seconds. Red diamonds: mean values. (B) Performance of activity recognition of random forest algorithms for 25 sensor configurations (best combination for “window length” and “number of features”). Green boxes: sensor configurations that were expected to perform well. Pink boxes: sensor configurations that were expected to perform poorly. Red diamonds: mean values. (C) Performance of activity recognition of random forest algorithms for 25 sensor configurations (“window length”: 20 seconds, “number of features”: best average rate of good prediction). Green and pink boxes: same chart as for panel B. [file peerj-08-10170-s004.png]
